# Assessment of Sonographic Ovarian Volume as a Potential Predictor of Polycystic Ovarian Syndrome (PCOS): A Hospital-Based Cross-Sectional Study

**DOI:** 10.2174/0118715303396709250721152449

**Published:** 2025-08-04

**Authors:** Babar Ali, Ibrahim Hadadi, Mohamed Adam, Wael Ali Faqihi, Mohammad Sayed, Sultan A Alotaibi, Awadia Gareeballah, Andrew England

**Affiliations:** 1 University Institute of Radiological Sciences and Medical Imaging Technology, Faculty of Allied Health Sciences, The University of Lahore, Lahore, Pakistan;; 2 Department of Radiological Sciences, College of Applied Medical Sciences, King Khalid University, Abha, Saudi Arabia;; 3 Department of Radiological Sciences, College of Applied Medical Sciences, Najran University, Najran, Saudi Arabia;; 4 Department of Radiological Sciences and Medical Imaging, College of Applied Medical Sciences, Majmaah University, Majmaah, Saudi Arabia;; 5 Department of Diagnostic Radiology Technology, College of Applied Medical Sciences, Taibah University, Al-Madinah Al-Munawwarrah, Saudi Arabia;; 6 The Discipline of Medical Imaging and Radiation Therapy, School of Medicine, University College Cork, Cork, Ireland

**Keywords:** Polycystic ovarian syndrome (PCOS), polycystic ovary disease (PCOD), infertility, body mass index, hirsutism, sonographic approach

## Abstract

**Introduction:**

Polycystic ovarian syndrome (PCOS) is a prevalent endocrine and metabolic disorder commonly associated with obesity and reproductive morbidities. The relationship between ovarian volume and BMI as well as ovarian morphology was therefore examined in this study to assess whether sonographic ovarian volume can serve as a supportive parameter in predicting PCOS in clinical practice.

**Methods:**

A hospital-based cross-sectional study of 100 women aged 20–40 years was conducted at the University of Lahore Teaching Hospital from December 2023 to September 2024. Participants were stratified according to normal weight (BMI < 25 kg/m^2^) and elevated BMI (BMI ≥ 25 kg/m^2^) groups with healthy and PCOS as subgroups within each group.

**Results:**

The left ovarian volume in PCOS patients was significantly larger than that in women without PCOS (mean 13.5 mL (SD 3.6) *vs* 10.4 mL (SD 1.9), *P* < 0.05). Likewise, the mean follicle size in the left ovary was also significantly larger in women with PCOS than in normal controls, 6.3 mm (SD 1.7) and 5.6 mm (SD 1.1), respectively (*P* < 0.05). PCOS showed a significant association with amenorrhea (85%) and acne (66%); however, 59% of participants showed hirsutism which was not significant.

**Discussion:**

The observed asymmetry in ovarian volume and the influence of BMI suggest underlying physiological variability. However, the absence of hormonal and metabolic data limits the interpretation, warranting cautious application of these findings.

**Conclusion:**

Ovarian volume alone is not a reliable diagnostic marker for PCOS. While the left ovary showed significant differences, the inconsistent findings support the need for a combined clinical, biochemical, and sonographic approach.

## 1 INTRODUCTION

Polycystic ovarian syndrome (PCOS) is one of the most common endocrine disorders in women of reproductive age [1], with a pooled mean prevalence of 21.27% [2]. Stein and Leventhal first diagnosed PCOS in 1935, and it is characterized by a spectrum of symptoms, including ovulatory dysfunction, hyperandrogenism, and polycystic ovarian morphology (PCOM) [3]. This disorder is a major public health concern because of its complicated etiology and multifaceted pathophysiology, which includes metabolic, hormonal, and genetic components. Women with PCOS frequently have a combination of menstrual irregularities, infertility, and symptoms of androgen excess, such as hirsutism and acne, in addition to metabolic changes like insulin resistance and elevated BMI [4, 5].

PCOS is a complex reproductive and metabolic disorder with lifelong health consequences. The diagnostic difficulties in PCOS are related to a complex clinical appearance and a lack of strict diagnostic criteria [6]. Recent international consensus statements, such as the 2018 International Evidence-Based Guideline for PCOS, have emphasized the need to standardize diagnostic thresholds for ovarian volume and antral follicle count to improve comparability across populations. Newer diagnostic strategies are also being explored, including anti-Müllerian hormone (AMH) as a biochemical proxy for follicle number and the use of three-dimensional and Doppler ultrasound to improve imaging accuracy. These developments highlight the evolving nature of PCOS diagnosis and the importance of continuing to evaluate traditional sonographic markers like ovarian volume across diverse clinical settings [7].

Transabdominal and transvaginal ultrasonography are important diagnostic techniques for PCOS because they allow for the non-invasive identification of PCOM [8-10]. Traditionally, PCOS was diagnosed based on clinical and biochemical indicators such as anovulation and increased androgen levels [11]. However, with the introduction of high-resolution ultrasonography, morphological criteria, particularly ovarian volume and follicle count—have become central to PCOS evaluation [12]. According to the Rotterdam criteria (2003), ovarian morphology is a crucial diagnostic element that must meet two of the three criteria (ovulatory dysfunction, hyperandrogenism, and PCOM). PCOM is indicated by these parameters, which include an ovarian volume of ≥10 cm^3^ or the presence of ≥12 follicles in one or both ovaries with a diameter of 2–9 mm [13, 14]. Despite its diagnostic value, the reliability of ovarian volume as a predictor of PCOS is still debated [15, 16]. Variations in ultrasound technology, operator experience, and patient demographics (age, ethnicity, and BMI) can all have an impact on ovarian volume measurement [17]. Recent research has questioned whether ovarian volume alone is enough to discriminate between PCOS and normal ovarian function. Although some research indicates that the degree of hyperandrogenism and ovulatory dysfunction may be correlated with ovarian volume, other studies show that women with and without PCOS have a considerable overlap in ovarian volume, especially, among those with elevated BMI [18-20].

Obesity, which affects 60-70% of women with PCOS, impairs the interpretation of ovarian volume. Women with elevated BMI (BMI ≥ 25 kg/m^2^) and PCOS have higher rates of insulin resistance than women with normal weight, which exacerbates the disorder's metabolic and reproductive symptoms. In addition to affecting ovarian morphology, this metabolic inefficiency puts afflicted women at risk for serious health consequences, such as type 2 diabetes, cardiovascular disease, and endometrial cancer [21-24]. The association between overweight/obesity and ovarian morphology highlights the importance of nuanced diagnostic approaches in PCOS that account for the interaction of metabolic and reproductive variables [25, 26]. Additionally, ovarian follicle size and dispersion have emerged as important criteria in determining PCOM. According to studies, while the total number of follicles in women with PCOS increases, their growth frequently stops prematurely, limiting the establishment of a dominant follicle and resulting in chronic anovulation [27, 28]. This altered folliculogenesis is important to the reproductive dysfunction seen in PCOS, contributing to infertility and an increased risk of miscarriage [29]. However, the extent to which follicle size and ovarian volume serve as independent predictors of PCOS is unknown, especially when stratified by BMI and other demographic characteristics [15, 25].

Therefore, this study aimed to assess the association between sonographic ovarian volume and PCOS status and to evaluate whether ovarian volume could serve as a supportive—not standalone—predictive parameter in the clinical assessment of PCOS. Using ovarian volume and follicle size, it seeks to evaluate the diagnostic value of ovarian morphology in the context of refining PCOS diagnostic criteria. Integrating ovarian morphology with clinical and metabolic parameters aims to overcome limitations in current diagnostic methods with the goal of increasing diagnostic accuracy, and subsequently, improving patient outcomes.

## 2 MATERIALS AND METHODS

### 2.1 Study Design and Sample Size

This cross-sectional study was conducted in the Radiology Department of the University of Lahore Teaching Hospital over a ten-month period (December 2023 - September 2024). A total of 100 women, aged 20 to 40, were recruited using a convenience sampling method. Ethical approval for this study was obtained from the Research Ethics Committee of the Faculty of Allied Health Sciences at the University of Lahore, approval number (Ref No: REC-UOL-1009-12-2023), and informed consent was obtained from all recruited patients. Each participant underwent a comprehensive clinical examination, followed by a transabdominal pelvic ultrasound. Additionally, demographic information, clinical history, and relevant laboratory investigations (where available) were gathered through structured interviews and a review of medical records. Participants were grouped based on BMI using a cutoff of 25 kg/m^2^, consistent with WHO guidelines for distinguishing normal weight from overweight/obesity, in order to assess the association between any form of elevated BMI and ovarian volume. Included participants were categorised into two groups based on PCOS status and BMI (Table **1**).

### 2.2 Sample Size Justification

The required sample size was calculated using the standard formula for prevalence studies:

n=Z^2^×P×(1−P)/d^2^

where Z is the Z-score corresponding to a 95% confidence level (1.96), P is the estimated prevalence of PCOS (21.27%), and d is the margin of error (10%). The prevalence estimate was derived from a systematic review by Deswal *et al*. (2020) [2], which reported a pooled mean PCOS prevalence of 21.27% across 27 international studies using different diagnostic criteria.

A 10% margin of error was selected based on guidance for exploratory studies, where larger margins are acceptable due to feasibility constraints and the descriptive nature of the study objectives [30]. Substituting these values yielded an estimated minimum sample size of approximately 65 participants. Our study included 100 participants, exceeding this minimum and providing sufficient data for exploratory subgroup analyses based on BMI and PCOS status.

### 2.3 Imaging Protocol

A standardised imaging methodology was used to ensure consistency and accuracy in data collection. All ultrasound exams were performed on a Toshiba Xario300 ultrasound machine with a 3-5 MHz convex transducer. To reduce inter-observer variability, all scans were performed by a single expert sonographer who was not aware of the participants' clinical diagnoses.

The study's participants were instructed to maintain a full bladder prior to the ultrasound examination to ensure proper visualisation of the pelvic organs. The individual was in a supine position during the transabdominal ultrasonography procedure. Initial evaluations involved examining the pelvic anatomy in general to rule out other possible diseases and the uterus for any anomalies. Using measurements taken in three orthogonal planes, the ellipsoid formula— ½ × length × breadth × height—was used to compute ovarian volume. The presence of 12 or more follicles (2–9 mm in diameter) or an increased ovarian volume (>10 cm^3^) in at least one ovary was used to define PCOS. Each ovary underwent a systematic follicle count, ensuring accurate identification and measurement while preventing overlap or duplication. All ultrasound scans were performed during the early follicular phase (days 2–5) in women with regular cycles. In women with irregular cycles, ultrasound was scheduled after excluding ovulation *via* endometrial thickness and clinical history. Key sonographic characteristics of PCOM, including the “string-of-pearls” appearance of follicles and stromal echogenicity, were carefully documented. To provide a comprehensive assessment, ovarian volume, follicle size, follicle count, and PCOM-specific sonographic features were recorded.

### 2.4 Statistical Analysis

Data were summarized using means and standard deviations, and ranges; categorical data were summarized as numbers and percentages. The frequencies of the quantitative variables were simply analysed through descriptive statistics. Comparisons between groups with respect to normally distributed numeric variables were performed using an independent t-test. *P* values <0.05 were considered statistically significant. A chi-squared test was performed to assess the association between some factors (*e.g.* hypertension, diabetes, infertility, obesity, hirsutism) and PCOS. Statistical analysis was performed using the Statistical Package for the Social Sciences (SPSS) Version 24.0.

## 3 RESULTS

The mean (SD) age of study participants was 24.9 (4.1) years. For the 50 females with PCOS, the mean (SD) age was 23.9 (3.2) years, whereas for the 50 females with normal ovaries, the mean (SD) age was 26.0 (4.6) years. Among them, 58% were married. Infertility was reported in 28% of participants (6% primary, 22% secondary), while 72% had no fertility issues. A summary of the included women’s characteristics are presented in Table **2** [31].

**Fig. (1):**
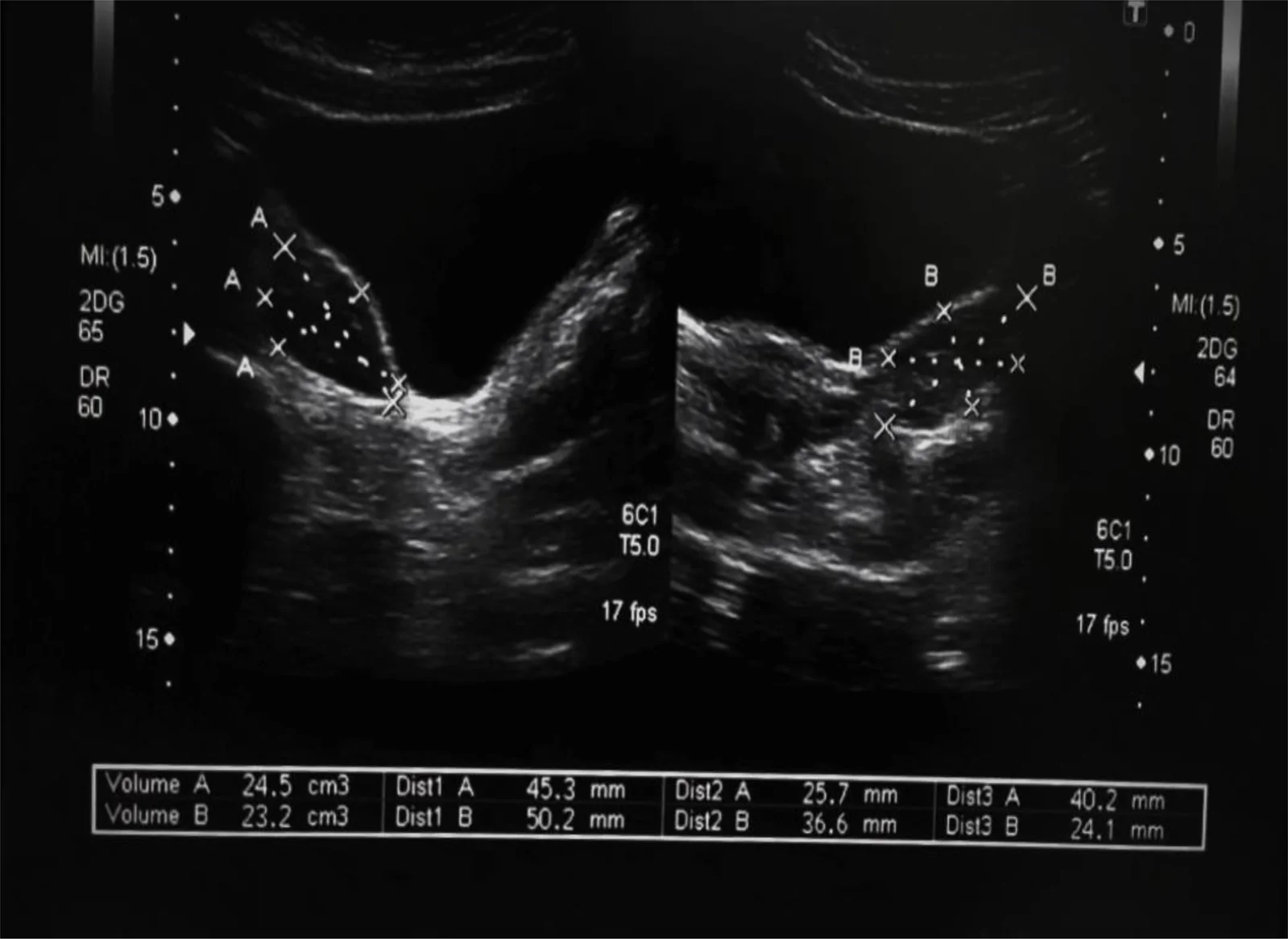
Transabdominal ultrasound of bilateral polycystic ovaries in a 27-year-old woman with high ovarian volume.

The mean (SD) right ovarian volume in PCOS participants was 11.2 (1.8) ml, and in normal women it was 11.4 (1.6) ml. However, in the left ovaries of PCOS participants, the mean (SD) ovarian volume was 13.5 (3.6) ml, and 10.4 (1.9) ml for normal females (Table **3**). Statistical significance was identified between the left ovarian volume in women with and without PCOS (*P*<0.05), whereas right ovarian volume differences between normal and PCOS women were not significant (*P*>0.05). This diversity is visible in ultrasound scans from study participants. For example, (Fig. **1**) illustrates an ultrasound image of a 27-year-old female (BMI ≥ 25 kg/m^2^) with bilateral polycystic ovaries and increased volumes of 24.5 cm^3^ and 23.2 cm^3^. Similarly, Fig. (**2**) shows a 22-year-old unmarried female with bilateral polycystic ovaries measuring 17 cm^3^ and 16.3 cm^3^.

The mean (SD) follicle size of the right ovary in females with PCOS was 6.8 (2) mm, and in women without PCOS, 6.1 (1.7) mm. However, in the left ovary of PCOS participants, the mean (SD) size of follicles was 6.4 (1.7) mm, whereas it was 5.6 (1.1) mm in normal females (Table **4**). Fig. (**3**) shows a 17-year-old female (BMI ≥ 25 kg/m^2^) with a unilateral polycystic ovary. Additionally, Fige. (**4**) shows bilateral polycystic ovaries in a 29-year-old unmarried female (BMI ≥ 25 kg/m^2^), with volumes of 13.6 cm^3^ and 14.6 cm^3^, which further reinforces the findings.

The mean (SD) follicle size of the right ovary in elevated BMI (BMI ≥ 25 kg/m^2^) participants was 6.4 (1.5) mm, and in normal-weight participants, it was 6.5 (2.2) mm. However, for the left ovaries of elevated BMI (BMI ≥ 25 kg/m^2^) participants, the mean (SD) size of follicles was 5.9 (1.3) mm, and 6.1 (1.7) mm in participants with normal weight (Table **5**).

PCOS was present in 55.4% of hypertensive women, 42.9% of diabetic women, and 54% of overweight/obese participants. Among clinical features, acne and amenorrhea showed significant associations with PCOS. Hirsutism, while more frequent in PCOS participants, was not found to be statistically significant. A detailed breakdown of these findings is presented in Table **6**.

## 4 DISCUSSION

In terms of the Integrated Estimate of PCOS, this study examined the correlation among ovarian morphometry, BMI, and clinical characteristics in PCOS. The results were that while PCOS women tended to have larger left ovarian volume and larger mean follicle size, there was no significant difference in right-sided measurements, suggesting lateral variability in ovarian changes. Moreover, patients with PCOS had a younger median age and a higher prevalence of obesity and hypertension than those without PCOS, suggesting that the characteristics of this condition are multifactorial.

The findings further strengthen the limitations of using ovarian volume alone as a diagnostic marker and highlight the importance of utilizing a combined approach of clinical, metabolic, and imaging data to achieve a more comprehensive diagnosis. The data also indicated that study participants with PCOS were relatively younger (mean age: 24.9±4.1) (n=100), which is consistent with the data used to derive the findings. This is consistent with earlier research by Gupta *et al*. and Saxena *et al*., who described PCOS as most commonly diagnosed between the ages of 20 and 30. These results highlight the need for early diagnosis and intervention during the reproductive years to facilitate better long-term health prognosis [32, 33].

In our study, we found a prevalence of hypertension in 56% of women diagnosed with PCOS, while a recent study has shown the prevalence of hypertension in Indian women with PCOS to be in the range of 22–29% [34, 35]. Although the analysis showed no statistically significant association between PCOS and hypertension, given the high prevalence, PCOS may be one of the factors that contribute to high blood pressure. This finding is in accordance with Gupta *et al*., whose study revealed that women with PCOS had a higher prevalence of hypertension (elevated BMI (BMI ≥ 25 kg/m^2^and normal) when compared with the general population [32]. Majumdar and Singh also recognized PCOS as a clinically diverse disorder contributing to an increased prevalence of health risks such as hypertension [36].

It is also clear that obesity is an ingredient that exaggerates this risk, as noted by Conway *et al*. [37] who observed increased systolic BP more frequently in PCOS women with elevated BMI (BMI ≥ 25 kg/m^2^), and Holte *et al*. showed a greater incidence of labile hypertension, which represents a prehypertensive state with the ability to disrupt metabolism [38]. The results in this study are in accordance with Wu *et al*., who reported a significantly higher risk of 1.62-fold for hypertension in patients with PCOS (incidence per 1,000 person-years was 7.85 for cases *vs* 4.23 for controls) [39]. These findings point to the cardiovascular danger of PCOS and highlight the need for early education and screening for blood pressure with preventive interventions to prevent long-term complications.

In our study, 35% of subjects were diabetic, and among them, 42.9% were diagnosed with PCOS. PCOS was present in 53.8% of non-diabetic women. Statistical analysis indicated an insignificant correlation between diabetes and PCOS in our cohort. Similar to our reports, many studies have demonstrated an independent association between PCOS and an elevated risk of type 2 diabetes mellitus. For example, Goodarzi *et al*. did not find an association between genetically predicted PCOS and diabetes risk, suggesting that obesity or elevated testosterone associated with PCOS may explain the association between PCOS and cardiometabolic diseases [40].

Moreover, an investigation conducted by Legro *et al*. has stated that women with PCOS are at a significantly increased risk of developing glucose intolerance, with prevalence rates for impaired glucose tolerance and diabetes of 31.1% and 7.5%, respectively [41]. Moreover, a meta-analysis conducted by Anagnostis *et al*. found that PCOS increases the risk for T2DM, which is further magnified when body weight is taken into account [42]. Differences between our results and other studies may result from sample size, population differences, and diagnostic criteria. The absence of an association found in our study suggests that there is merit in a larger population and a more diverse sample in order to provide clarification on the relationship between diabetes and PCOS. Nevertheless, due to the strong evidence from the other studies, clinicians should continue to assess glucose metabolism in women with PCOS, particularly those with additional risk factors (*e.g.* obesity), to enable early recognition and treatment of glucose intolerance and diabetes.

We also found that 85% of study participants experienced amenorrhea, emphasizing the major effect of PCOS on menstrual health. This finding is consistent with earlier study that reported menstrual irregularities in 95% of PCOS women, and similar rates of 96.6 and 92.8% in elevated BMI and lean women respectively [33]. Additionally, Siddiqui *et al*. reported menstrual irregularities in all PCOS patients diagnosed with hyperandrogenism and pelvic ultrasonography [43]. Such studies highlight the need to recognize menstrual disturbances as a core presenting feature of PCOS that must be evaluated and managed effectively.

Our study showed that 59% of PCOS participants had hirsutism, and 66% had experienced acne, with acne being significantly associated with PCOS, suggesting the common dermatological manifestations of the condition. These results are in agreement with past studies that have demonstrated a very high prevalence of dermatological manifestations within this population [44, 45]. Due to the significant effect these symptoms have, dermatological assessment should be part of the diagnosis and management strategy for PCOS. Addressing these issues early on can improve patient outcomes and overall quality of life. The absence of significant associations for the right ovary, hirsutism, and diabetes may reflect population-specific variability or limited sample size. It is also possible that factors like hormonal control, phenotype variation, or misclassification may have masked associations. These null results emphasize the heterogeneity of PCOS and highlight the importance of using multiple diagnostic indicators.

Our study also demonstrated that the mean ovarian volume of the right ovary in PCOS participants was 11.2 mL, compared to 11.4 mL in non-PCOS individuals, with no significant difference observed. However, the left ovarian volume was significantly larger in those with PCOS (13.5 mL) than in those without (10.4 mL). This asymmetry aligns with the findings of Mansour *et al*., who reported that increased ovarian volume is an indicator of hyperandrogenemia state in females with PCOS [15]. Such findings confirm that ovarian volume, particularly of the left ovary, is a distinguishing feature in the diagnosis of PCOS. The present study highlights the lateral differences and supports the need for bilateral examination of the ovaries during ultrasonography to enhance diagnostic accuracy and provide a comprehensive evaluation of ovarian morphology. The left ovary is known to have different venous drainage, typically *via* the left renal vein, which may influence ovarian perfusion, hormone exposure, and follicular activity [46, 47]. Some studies have reported a dominance in ovulation on the right side, possibly due to higher vascular resistance on the left, which could result in greater follicular accumulation in the left ovary over time [48, 49]. These anatomical differences may partly explain the asymmetric findings observed in this study and in other PCOS cohorts. However, clinical implications remain speculative, and further studies using Doppler imaging or longitudinal ovulatory tracking may provide more insight into lateral ovarian physiology in PCOS.

For those with PCOS, mean follicle size in the right ovary was 6.8 mm, while for those without PCOS it was 6.1 mm, implying that no statistically significant difference was observed. The average size of the follicles in the left ovary in the PCOS group was 6.4 mm and 5.6 mm in the non-PCOS group, with a statistically significant difference. This finding is consistent with previous studies demonstrating that polycystic ovaries are identifiable by a greater follicle size and increased number. As an example, one work by Jonard *et al*. found that the mean follicle number per ovary (FNPO) of 2–5 mm follicles was found to be significantly greater in polycystic ovaries than in controls, while that of 6–9 mm follicles did not differ between groups [50].

It indicates that the accumulation of smaller follicles is a distinguishing feature of polycystic ovaries. The clinical implications are the need to perform detailed ultrasonographic evaluation of both the ovaries when patients are being assessed for PCOS. In view of the noted asymmetry in follicle size, using measurements from only one ovary can falsely underdiagnose or misdiagnose the condition. Consequently, the addition of bilateral ovarian evaluations to standard diagnostic methodologies might improve the specificity of PCOS diagnoses and facilitate appropriate management for patients with the condition.

There are some limitations that should be acknowledged in this study. It was performed in a single center setting, limiting the ability to generalize the current findings to the wider population. Second, the cross-sectional design constrains the inference of causality between ovarian morphology and clinical or metabolic markers. Third, the imaging by a single sonographer lowered inter-observer variability in ultrasound measurements but does not exclude possible measurement bias. Fourth, the lack of biochemical marker comparison, such as serum androgens, LH/FSH ratio, or insulin resistance indices, limited the analysis. These would have allowed for a more comprehensive assessment of PCOS phenotypes. Future studies should integrate biochemical and imaging parameters to enhance diagnostic accuracy. These limitations should be addressed in future research utilizing long-term as well as multi-center cohorts for a more powerful and comprehensive assessment.

This study focused on assessing the association between BMI, ovarian volume, and PCOS status using exploratory analysis and descriptive comparisons. While this approach was suitable for the study’s scope, future research should employ multivariate analysis techniques to control for confounding variables such as age, hormonal levels, and metabolic comorbidities. Incorporating such factors into adjusted models may improve diagnostic precision and better isolate the individual contribution of ovarian morphology to PCOS risk.

## CONCLUSION

This study aimed to assess whether ultrasound-determined ovarian volume could serve as a supportive parameter in predicting PCOS. Our findings show that while there was a significant difference in left ovarian volume between women with and without PCOS, no difference was observed in the right ovary. These results highlight the limitations of relying on ovarian volume alone as a diagnostic marker for PCOS. A holistic diagnostic approach that combines clinical, biochemical, and ultrasonographic parameters remains essential for accurate diagnosis. Future studies should explore the role of novel imaging technologies and multidimensional diagnostic strategies, particularly in women from diverse ethnic backgrounds and phenotypic presentations.

## Figures and Tables

**Fig. (2):**
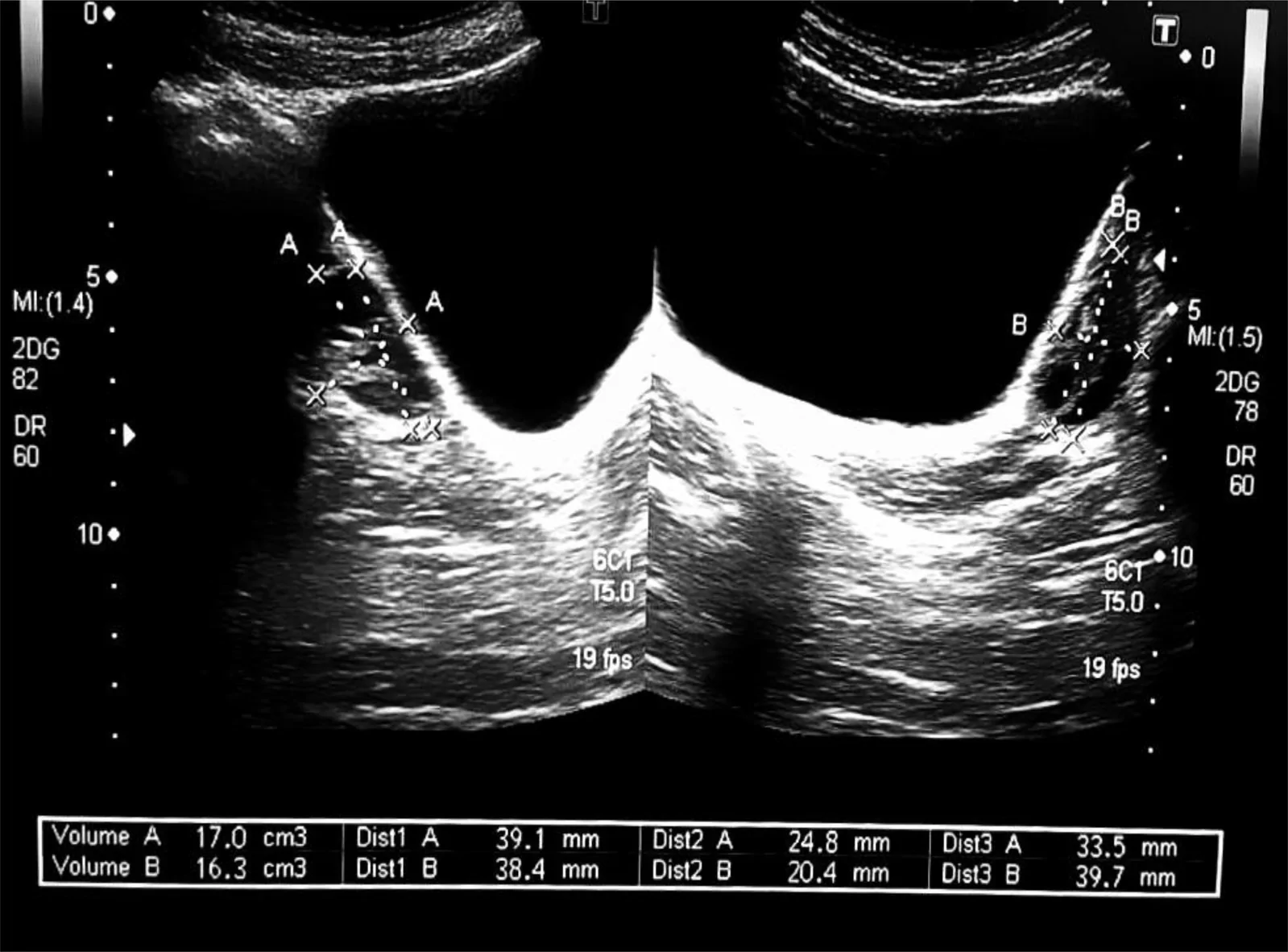
Ultrasound image of bilateral PCOS in a 22-year-old woman with moderate ovarian volume.

**Fig. (3):**
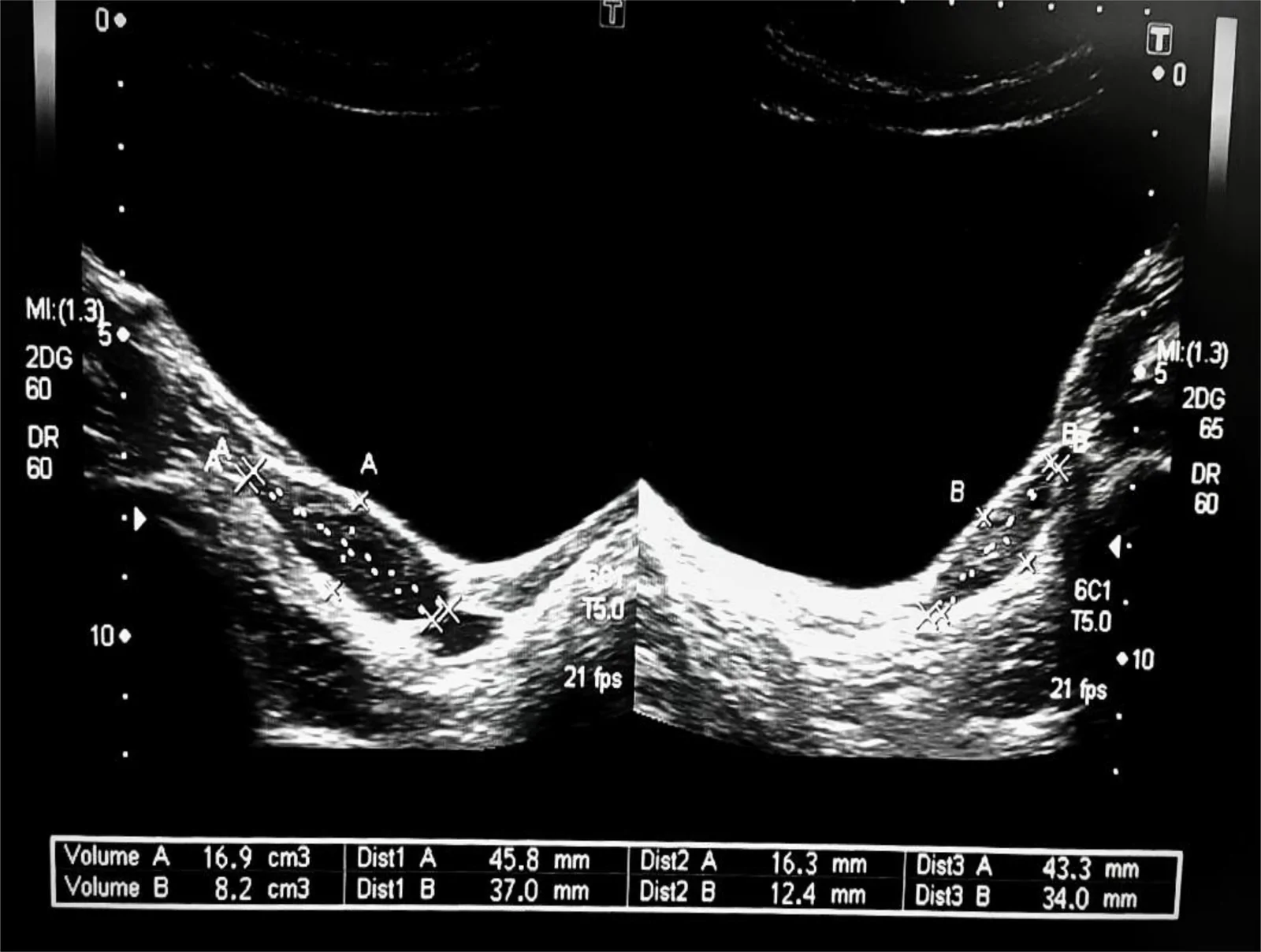
Unilateral polycystic ovary in a 17-year-old adolescent with elevated BMI.

**Fig. (4):**
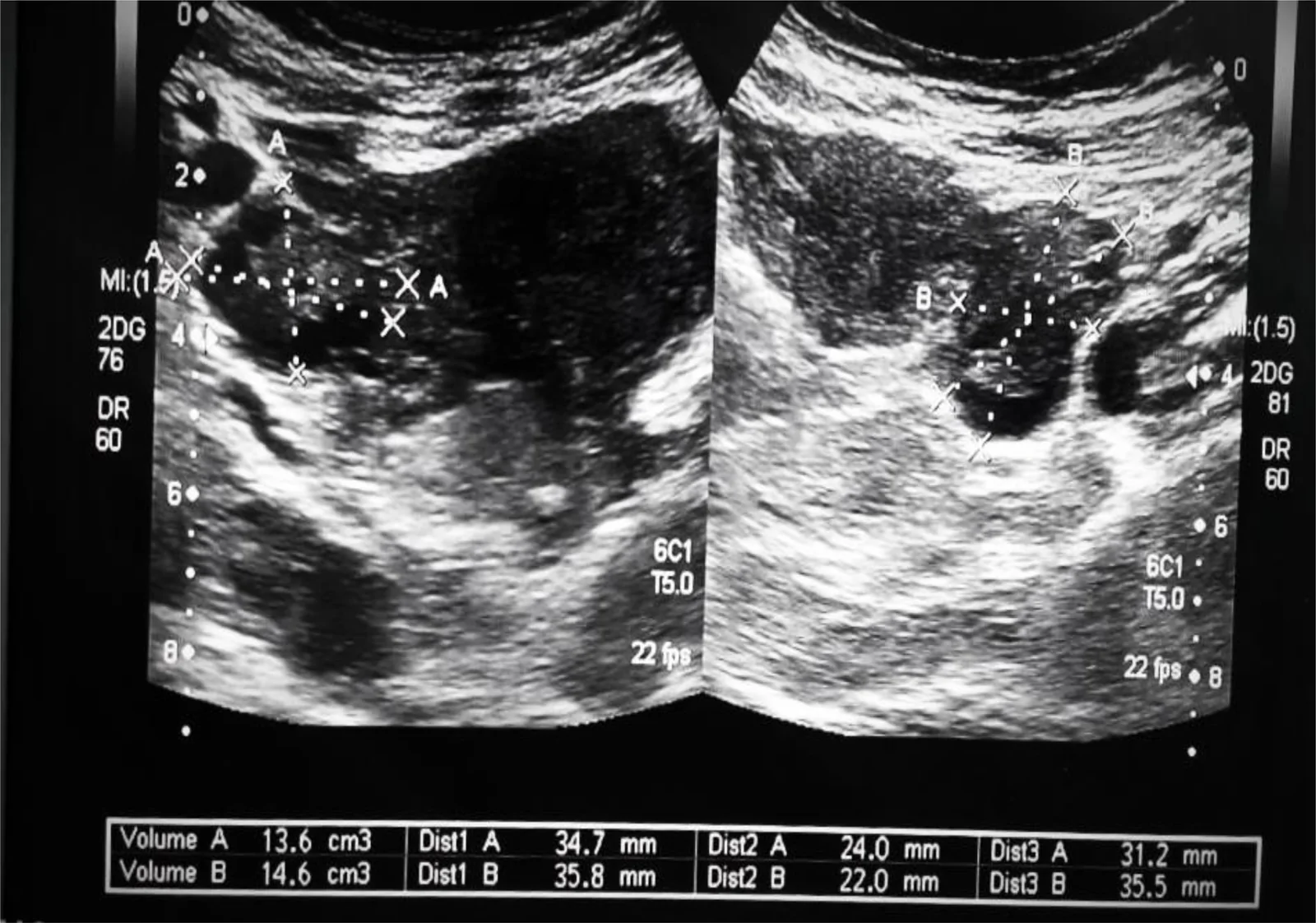
Bilateral polycystic ovaries in a 29-year-old woman with elevated BMI.

**Table 1:** Summary of study's inclusion and exclusion criteria.

**Inclusion Criteria**
Group 1: Women with BMI < 25, including both healthy individuals and those diagnosed with PCOS. Group 2: Women with BMI ≥ 25, including both healthy individuals and those diagnosed with PCOS.
**Exclusion Criteria**
Females with treated PCOS Infertility treatment Congenital anomalies Ovarian abnormalities other than PCOS

**Table 2:** Characteristics of women with PCOS and without PCOS included in the study.

**Variable**		**PCOS**	**Frequency**
**Hirsutism**		Yes	59
		No	41
**Overweight/Obesity (BMI ≥ 25 kg/m^2^)**		Yes	50
		No	50
**Acne**		Yes	66
		No	34
**Hypertension**		Yes	56
		No	44
**Diabetes**		Yes	35
		No	65
**Amennorhea**		Yes	85
		No	15
**String like appearance**		Yes	50
		No	50
**Central echogenic stroma**		Yes	50
		No	50

**Note:** Hirsutism was evaluated using the modified Ferriman-Gallwey (mFG) score. Nine androgen-sensitive body areas were examined, and each area was graded from 0 (no hair) to 4 (frank virilization). A total score ≥8 was used to define clinically significant hirsutism [31].

**Table 3:** Summary of ovarian volume statistics between participant groups.

-			**Ovarian Volume (mL)**		
**Ovary**	**PCOS**	**NO.**	**Mean**	**SD**	***P* Value**
**Right**	Present	50	11.2	1.8	0.650
	Absent	50	11.4	1.6	
**Left**	Present	50	13.5	3.6	**<0.001**
	Absent	50	10.4	1.9	

**Table 4:** Summary of ovarian follicle statistics between participant groups.

-			**Ovarian Follicle Size (mm)**		
**Ovary**	**PCOS**	**NO.**	**Mean**	**SD**	***P* Value**
**Right**	Present	50	6.8	2.0	0.060
	Absent	50	6.1	1.7	
**Left**	Present	50	6.4	1.7	**<0.012**
	Absent	50	5.6	1.1	

**Table 5:** Summary of ovarian follicle statistics between obesity groups.

-			**Ovarian Follicle Size (mm)**		
**Ovary**	**BMI ≥ 25 kg/m^2^**	**NO.**	**Mean**	**SD**	***P* Value**
**Right**	Yes	50	6.4	1.5	0.777
	No	50	6.5	2.2	
**Left**	Yes	50	5.9	1.3	0.604
	No	50	6.1	1.7	

**Table 6:** Association of selected clinical features and metabolic comorbidities with PCOS status.

-		**PCOS**		
		**Present**	**Absent**	**Total**
Hypertension*	**Present**	31 (55.4%)	25 (44.6%)	56
	**Absent**	19 (43.2%)	25 (56.8%)	44
	**Total**	50	50	100
Diabetes*	**Present**	15 (42.9%)	20 (57.1%)	35
	**Absent**	35 (53.8%)	30 (46.2%)	65
	**Total**	50	50	100
Overweight/Obesity* **(BMI ≥ 25 kg/m^2^)**	**Present**	27 (54.0%)	23 (46.0%)	50
	**Absent**	23 (46.0%)	27 (54.0%)	50
	**Total**	50	50	100
Hirsutism*	**Present**	24	35	59
	**Absent**	26	15	41
	**Total**	50	50	100
Acne**	**Present**	33	33	66
	**Absent**	17	17	34
	**Total**	50	50	100
Amenorrhea**	**Present**	48	37	85
	**Absent**	2	13	15
	**Total**	50	50	100

**Note:** *Chi-squared test is not significant (*P*>0.05)

**Chi-squared test is significant (*P*<0.05)

Clinical symptoms (*e.g.*, acne, hirsutism, amenorrhea) represent manifestations of PCOS, not causative risk factors. Variables are reported for associative analysis only.

## Data Availability

The datasets used and analyzed during the current study are available from the corresponding author upon reasonable request.

## LIST OF ABBREVIATIONS

PCOS: Polycystic Ovary Syndrome
BMI: Body Mass Index
PCOM: Polycystic Ovarian Morphology
SD: Standard Deviation

## STANDARDS OF REPORTING

STROBE guidelines were followed.
